# Consumers’ second-hand clothing purchase intention: integrating TPB, face consciousness and past purchase experience

**DOI:** 10.3389/fpsyg.2025.1645116

**Published:** 2025-10-13

**Authors:** Qi Zhou, Xiaofen Ji, Nan Li, Liyuan Zhou

**Affiliations:** ^1^School of Art and Design, Zhejiang Sci-Tech University, Hangzhou, China; ^2^College of Art, Jiujiang University, Jiujiang, China; ^3^International Institute of Fashion Technology, Zhejiang Sci-Tech University, Hangzhou, China; ^4^China National Silk Museum, Hangzhou, China; ^5^School of Design and Fashion, Zhejiang University of Science and Technology, Hangzhou, China; ^6^Dougou Central Primary School, Wuhu, China

**Keywords:** face consciousness, integrated framework, second-hand clothing, purchase experience, purchase intention

## Abstract

This study establishes an integrated theoretical framework to comprehensively examine the factors influencing Chinese consumers’ second-hand clothing (SHC) purchase intention. Utilizing a randomly sampled dataset of 654 Chinese consumers, structural equation modeling (SEM) was conducted using AMOS software. The analysis demonstrates that our integrated model exhibits superior predictive power for SHC purchase intention compared to existing frameworks, achieving statistically satisfactory results. The findings provide support for all hypothesized relationships among the theoretical constructs, while the significant mediating effects of the proposed mediators were also confirmed. Notably, invariance tests revealed the moderating effect of past purchase experience.

## 1 Introduction

The fast fashion industry has undergone rapid expansion in recent years, with companies increasingly accelerating production cycles to stimulate consumer purchasing frequency (Niinimäki et al., 2020). A prime example of this phenomenon is the Chinese market, where the emergence of ultra-fast e-commerce brands such as Shein has been facilitated by highly responsive, on-demand supply chains. These operational models enable the daily release of thousands of new products, thereby directly contributing to an unprecedented acceleration of consumption patterns. As the world’s largest textile producer and apparel market (Lang et al., 2019), Chinese fashion industry presents significant environmental challenges. The textile industry is regarded as one of China’s three most environmentally harmful sectors, contributing significantly to pollution through the discharge of wastewater, the emission of airborne pollutants, and the generation of toxic textile waste (Bjurbäck, 2015). Globally, over 30 million tons of clothing are discarded annually, creating severe resource depletion and urban pollution (Rausch and Kopplin, 2021). Furthermore, the fashion industry accounts for approximately 10% of global carbon emissions (Ro, 2020), exacerbating climate change concerns.

In response to these environmental impacts, growing consumer interest in sustainable alternatives has emerged, particularly in SHC consumption (Gopalakrishnan and Matthews, 2018). Academic research suggests that SHC adoption can significantly reduce textile waste and environmental pollution (Khurana and Tadesse, 2019; Machado et al., 2019). Chinese substantial population and dominant position in global apparel consumption make it a critical market for SHC growth (Su et al., 2019), with both environmental and economic benefits for sellers (Armstrong and Park, 2020).

Existing research on SHC consumption has predominantly employed Western-centric behavioral theories, particularly the Theory of Planned Behavior (TPB), to examine purchase intention (Kian et al., 2022; Madeline et al., 2023; Tryphena and Arul Aram, 2023). While TPB effectively predicts intention through attitude, perceived behavioral control (PBC), and subjective norm (Ajzen and Driver, 1991), significant gaps remain in understanding SHC adoption within specific cultural contexts. Previous studies have primarily focused on psychological and situational factors, often neglecting important cultural dimensions and moderating effects.

A critical oversight in existing literature is the inadequate consideration of face consciousness–a culturally salient construct in collectivist societies like China (Bao et al., 2003; Hwang, 2006). Prior research has either treated face consciousness unidimensionally or examined only its positive aspects (Li and Cui, 2021; Liu et al., 2021), failing to account for its potential inhibitory effects (e.g., stigma avoidance). The existing literature has examined several moderating factors influencing SHC purchase intention. Kian et al. (2023) established that prior purchase experience significantly moderates the relationship between perceived risks and SHC consumption. Similarly, Zahid et al. (2023) identified self-engagement as a critical moderator in the association between mindful consumption and SHC adoption. Further investigations have revealed that both generational differences (Liang and Xu, 2018) and cross-cultural variations (Xu et al., 2014) serve as important moderating factors affecting various determinants of SHC purchase behavior. Most recently, Kian et al. (2024) demonstrated that aesthetic risk moderates the impact of sanitary risk on purchase intention. However, despite these valuable contributions, a comprehensive examination of culturally specific and experience-based moderating variables within the SHC context remains absent from the extant literature.

This study addresses these gaps through three primary objectives. First, by adopting the TPB as the guiding framework, this study incorporates desire for gaining face, fear of losing face, and past purchase experience to develop a more robust model for understanding the SHC purchase intention of Chinese consumers. Second, the proposed model is empirically compared with the original TPB model to assess its predictive superiority. Third, the mediating and moderating roles of the identified research variables are examined to further clarify their influence on SHC purchase intention among Chinese consumers. By incorporating psychological, cultural, and experiential factors, this research offers a more comprehensive model for understanding SHC purchase intentions in culturally specific contexts.

This study contributes to the extant literature by integrating cultural constructs, specifically face consciousness, and past purchase experience into the TPB. This extended integrative model is proposed to investigate the cumulative effects of these factors, thereby confirming the utility of the proposed framework within the domain of SHC. The empirical findings provide actionable insights for marketing practitioners and offer a strategic blueprint for the enhancement of SHC purchase intentions.

## 2 Literature review

### 2.1 Theory of planned behavior

Theory of Planned Behavior was first developed by Ajzen and Driver (1991). At its core, TPB incorporates behavioral intention as a key determinant of action (Ajzen and Driver, 1991), reflecting an individual’s conscious decision-making or planned motivation. According to TPB, purchase intention and behavior can be accurately predicted through three primary factors: attitude, PBC, and subjective norm (Ajzen and Driver, 1991; Ajzen, 2002). In recent years, the TPB has been widely applied across various domains of consumer behavior research, including studies on sustainable clothing and SHC (Kian et al., 2022; Madeline et al., 2023; Tryphena and Arul Aram, 2023). Furthermore, its significant utility has also been demonstrated within pro-environmental contexts, such as the adoption of conservation agriculture practices (Valizadeh et al., 2023, 2024) and water conservation behaviors. In conclusion, the TPB has proven to be a valuable framework for analyzing the behavioral intentions of diverse populations across a wide range of disciplines. However, the theory overly relies on rational decision-making while neglecting the influence of cultural factors and emotions in green consumption contexts (ElHaffar et al., 2020).

According to Ajzen and Driver (1991), attitude is defined as the extent to which an individual holds a favorable or unfavorable evaluation regarding a specific behavior. When consumers perceive SHC consumption as socially beneficial and morally correct, they are more likely to develop positive purchase intention. Empirical evidence consistently demonstrates that attitude serves as a significant predictor of purchase intention in sustainable fashion and SHC contexts. Kian et al. (2022) identified attitude as a robust determinant of SHC purchase intention, while Madeline et al. (2023) confirmed its predictive power specifically among young Australian female consumers. Beyond consumer behavior contexts, the critical role of attitude has been demonstrated across a diverse range of fields and population groups. For instance, Mohammadzadeh et al. (2025) identified a significant positive impact of stockmen’s attitude toward pro-animal behavior on their subsequent behavioral manifestation. Similarly, Valizadeh et al. (2023) established that farmers’ attitude toward water conservation significantly influenced both their corresponding intention and actual conservation behavior. Collectively, these findings underscore the broad applicability of the attitude construct in predicting and understanding behavior across disparate domains.

Subjective norm, as conceptualized by Ajzen and Driver (1991), represents an individual’s perception of social pressure to perform or avoid a particular behavior, influenced by the attitudes of significant others (Ajzen and Fishbein, 1980). When individuals perceive that important referent groups approve of SHC consumption, this social influence serves as a powerful motivator for behavioral intention. Extensive empirical research has established the significant role of subjective norm in shaping purchase intention within sustainable fashion contexts. Kian et al. (2022) identified a positive correlation between subjective norm and SHC purchase intention, while Sobuj et al. (2021) specifically demonstrated this normative influence among young Bangladeshi consumers. The robustness of the subjective norm construct is further corroborated by recent research within environmental domains. For instance, a strong influence of farmers’ subjective norms regarding water conservation on their adoption decisions was identified by Valizadeh et al. (2023). This finding is further supported by a meta-analysis conducted by Morren and Grinstein (2021), which confirmed that subjective norm is a consistent predictor of pro-environmental behavior across diverse cultural contexts.

Perceived behavioral control is conceptually defined as an individual’s self-assessment of the ease or difficulty associated with executing a specific behavior (Ajzen and Madden, 1986). Empirical evidence suggests that consumers’ perceived level of control over SHC purchases is positively correlated with their purchase intention. Within the domain of sustainable fashion research, PBC has been consistently identified as a significant determinant of consumer behavior. This relationship was substantiated by Chaturvedi et al. (2020), who demonstrated PBC’s strong predictive power regarding sustainable clothing purchase intentions. Further validation was provided by Kian et al. (2022), whose findings confirmed the positive effect of PBC on SHC consumption decisions. The relevance of PBC is also demonstrated within the domain of environmental conservation behaviors. Recent research by Valizadeh et al. (2024) established that among Iranian landowner farmers, PBC significantly predicted the intention to apply conservation agriculture technologies and practices. Furthermore, PBC was identified as a stronger predictor of this intention than either attitude or subjective norm, thereby reinforcing the cross-contextual validity of this critical component of the TPB. Taking these factors into account, we propose that:

H1: Attitude positively affects consumers’ SHC purchase intention.

H2: Subjective norm positively affects consumers’ SHC purchase intention.

H3: PBC positively affects consumers’ SHC purchase intention.

### 2.2 Face consciousness

Face consciousness (Mianzi consciousness), deeply rooted in Confucian cultural values, represents a critical factor influencing consumer behavior in collectivist societies like China (Huang et al., 2019). This construct refers to an individual’s concern for maintaining social prestige and reputation (Li and Wang, 2020), which subtly shapes consumption patterns. Consumers with high face consciousness actively seek to enhance their social standing while avoiding behaviors that might cause embarrassment or loss of status (Li et al., 2015). Given the paramount importance of social perception in Chinese culture, face consciousness emerges as a crucial determinant of purchasing decisions.

In recent years, research has begun exploring how face consciousness influences sustainable consumption behaviors (Wei and Jung, 2017; Chen K. et al., 2019; Wei and Shen, 2025). Recent research has revealed contradictory findings regarding face consciousness’s impact on sustainable consumption. While some studies demonstrate positive associations with sustainable fashion adoption (Wei and Jung, 2017) and new energy vehicle purchases (Chen K. et al., 2019), others show negative correlations with energy-saving behaviors or connections to conspicuous consumption (Wang et al., 2018). These discrepancies likely stem from the conventional unidimensional treatment of face consciousness in their research context which fails to distinguish between its two fundamental aspects: desire for gaining face and fear of losing face (Zhang et al., 2011).

The desire for gaining face reflects individuals’ motivation to enhance their social standing, while fear of losing face represents anxiety about social disapproval (Kopania, 2020). These distinct dimensions involve separate psychological processes (Wei and Shen, 2025) and yield divergent consumption outcomes. Within SHC context, a positive purchase intention can be cultivated by the perception that SHC symbolizes environmentally friendly attributes (Inhwa et al., 2021) or a distinctive taste (Machado et al., 2019), as these perceptions are associated with face-gaining behaviors. Conversely, a negative purchase intention can be triggered by associations with financial constraints or hygiene concerns (Inhwa et al., 2021), which are perceived as face-losing behaviors. The significant influence of face consciousness on consumers’ continued purchase intention for organic food has been empirically confirmed by Qi et al. (2023), underscoring its relevance to the SHC domain.

According to TPB, attitude is established as a primary antecedent of behavioral intention (Ajzen and Driver, 1991). It is posited that face consciousness serves as a formative factor in shaping attitude toward SHC. Consumers with a high desire for gaining face are likely to evaluate SHC more favorably, as these products are perceived as effective instruments for facilitating the attainment of valued social goals, such as gaining respect or winning social approval. Conversely, consumers with a high fear of losing face tend to evaluate SHC more negatively, based on the perception that such products could potentially diminish their social standing, for instance, by damaging their reputation. These subsequently formed attitudes are then translated into corresponding purchase intentions. This mediating role of attitude is supported by the work of Long and Abd Aziz (2022), who found that face gaining exerts an indirect impact on behavioral intention through attitude. Furthermore, Li and Su (2007) established that face consciousness, representing both social reputation and status identity, significantly impacts consumption attitudes and purchase intentions.

Face consciousness assumes particular significance in Confucian collectivist cultures, where social norms serve as fundamental determinants of individual behavior (Kim and Nam, 1998; Jin and Kang, 2010; Cho et al., 2013). This psychological construct plays a pivotal role in maintaining social norms, as individuals with heightened face consciousness demonstrate greater sensitivity to the social impressions created by their actions (Zhao et al., 2019). Within Confucian relational frameworks (Bao et al., 2003; Hwang, 2006), such individuals consistently prioritize external evaluations over personal preferences when making consumption decisions.

The theory of reasoned action (Ajzen and Fishbein, 1977) provides a theoretical foundation for understanding how subjective norms - defined as perceived social pressures from significant referent groups to perform or not perform a behavior- influence behavioral intentions. The consumption of SHC is often strongly governed by such norms (Kian et al., 2022). As face consciousness is inherently linked to the perception of social expectations, individuals with strong face consciousness are considered to be particularly susceptible to these normative influences (Bao et al., 2003) and are frequently motivated to engage in pro-social behaviors when perceived social pressures exceed specific thresholds (Leary and Kowalski, 1990). This relationship is further elucidated by Hawkins et al. (2020), who demonstrated that face consciousness operates as a cultural value that primarily shapes consumer behavior through normative mechanisms. Supporting this view, Long and Abd Aziz (2022) found that face gaining exerts an indirect impact on outbound travel intention through subjective norm, a finding that underscores the broader applicability of this normative pathway. Based on the above theoretical synthesis, we propose the following hypotheses:

H4: Desire for gaining face positively affects consumers’ SHC purchase intention.

H5: Fear of losing face negatively affects consumers’ SHC purchase intention.

H6: Desire for gaining face indirectly affects consumers’ SHC purchase intention through attitude.

H7: Fear of losing face indirectly affects consumers’ SHC purchase intention through attitude.

H8: Desire for gaining face indirectly affects consumers’ SHC purchase intention through subjective norm.

H9: Fear of losing face indirectly affects consumers’ SHC purchase intention through subjective norm.

### 2.3 Past purchase experience

Existing research has demonstrated important limitations in examining the TPB variables in isolation. While Kian et al. (2022) investigated the effects of TPB constructs on SHC purchase intention, they failed to account for consumers’ past purchase experience. This oversight is significant given that Hur (2020) established fundamental differences in decision-making processes between existing and potential SHC consumers. The incorporation of past behavior into the TPB framework has been shown to enhance predictive accuracy regarding behavioral intentions (Chien et al., 2012), as demonstrated across various consumption contexts (Cheng et al., 2005; Hsieh et al., 2016).

Past experience serves as a critical determinant of behavioral intentions, reflecting consumers’ familiarity with specific consumption behaviors. Research has identified both direct and indirect effects of past experience on intention formation (Liu et al., 2020), though the moderating mechanisms remain underexplored (Hsieh et al., 2016; Chen X. Y. et al., 2019). Understanding these moderating effects holds particular importance for marketing practitioners, as past experience represents an exogenous variable that necessitates differentiated communication strategies for experienced versus inexperienced consumer segments.

Empirical evidence supports the moderating role of past experience in shaping the relationships between TPB variables and behavioral intentions. Hsieh et al. (2016) demonstrated that prior visit experience moderated the effects of attitude and PBC on intentions among Taiwanese youth. Similarly, Kidwell and Jewell (2008) found that past experience amplified the influence of subjective norms and PBC on behavioral intentions. Based on this theoretical foundation, we propose the following hypotheses:

H10a: Past purchase experience moderates the relationship between attitude and SHC purchase intention.

H10b: Past purchase experience moderates the relationship between subjective norm and SHC purchase intention.

H10c: Past purchase experience moderates the relationship between PBC and SHC purchase intention.

**FIGURE 1:**
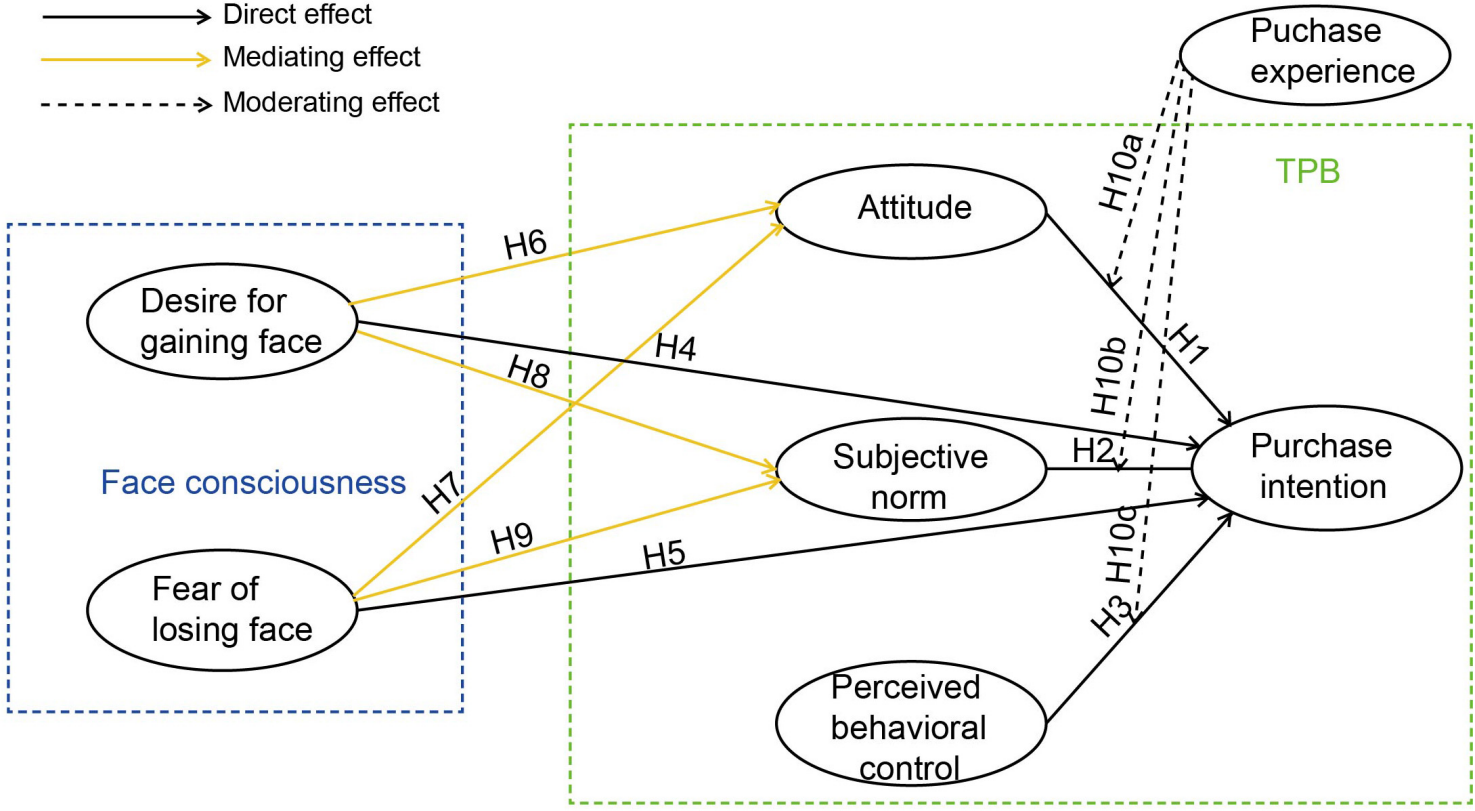
Comprehensive model.

Building upon this theoretical analysis, we present an integrated conceptual framework (Figure 1) that incorporates past purchase experience as key moderating variables.

## 3 Methodology

### 3.1 Question design

The research instrument comprised two distinct sections designed to systematically collect relevant data. The first section captured participants’ demographic characteristics and past purchase experience with SHC. The second section incorporated validated measurement scales utilizing a 5-point Likert format, assessing the following constructs: TPB variables (attitude, subjective norm, and PBC), Face consciousness (desire for gaining face, fear of losing face), SHC purchase intention. All measurement items were adapted from established instruments in prior studies (Ajzen, 2002; Zhang et al., 2011; Chu and Chen, 2016; Borusiak et al., 2020; Denley et al., 2020; Nie et al., 2020; Kim et al., 2022), with their reliability and validity having been previously verified. Furthermore, two attention-check questions were embedded within the survey instrument to screen for inattentive respondents and to safeguard the integrity and reliability of the collected data. The questionnaire underwent a rigorous translation and validation process: (1) Bilingual researchers performed bidirectional translation (English-Chinese-English) to ensure linguistic equivalence; (2) Four doctoral experts reviewed the instrument and provided feedback; (3) Twenty randomly selected participants completed a pilot test, leading to refinement of ambiguous items. The final measurement items are presented in Table 1.

**TABLE 1:** Scale items.

Construct	Item	References
Desire for gaining face	DGF1: i can show my social responsibility of environmental protection to others by purchasing SHC.	Zhang et al., 2011
	DGF2: i can win much more social approval by purchasing SHC.	
	DGF3: i can improve people’s impression of me by buying SHC.	
Fear of losing face	FLF1: i worry that others will look down upon me if I purchase SHC.	Zhang et al., 2011; Silva et al., 2021
	FLF2: i’m afraid of being criticized by others for purchasing SHC.	
	FLF3: i avoid purchasing SHC because it might damage my reputation.	
Perceived behavioral control	PBC1: it is on my own will to purchase SHC.	Chu and Chen, 2016; Nie et al., 2020
	PBC2: i have the opportunity and resources to purchase SHC.	
	PBC3: i possess the knowledge and ability to purchase SHC.	
	PBC4: i can purchase SHC without any help.	
Attitude	ATT1: it is good to purchase SHC.	Ajzen, 2002
	ATT2: it is pleasant to purchase SHC.	
	ATT3: it is valuable to purchase SHC.	
	ATT4: it is beneficial to purchase SHC.	
Subjective norm	SN1: my family support me to purchase SHC.	Denley et al., 2020; Nie et al., 2020
	SN2: my classmates support me to purchase SHC.	
	SN3: my teachers support me to purchase SHC.	
	SN4: the mass media support me to purchase SHC.	
Purchase intention	PI1: i’m very likely to purchase SHC in the future.	Borusiak et al., 2020
	PI2: certainly, i will purchase SHC.	

### 3.2 Sample

The study focused on Chinese consumers as the target population. Data collection was conducted through an online survey platform^1^ from January to March 2024. This platform facilitated random sampling procedures as demonstrated in previous research (Lu et al., 2019). To enhance the representativeness of the sample of Chinese consumers, a stratified random sampling method was employed, utilizing geographic region as the stratification variable. Mainland China was divided into several major regions (e.g., Northern, Southern, Eastern, Western, and Central). Quotas were established for each geographic stratum. This approach was designed to ensure the sample was drawn from a diverse array of geographic clusters, thereby improving its national representativeness. To improve participation rates, a nominal incentive of 2 RMB, distributed via digital red envelopes, was offered to respondents upon the survey’s completion.

Participants were explicitly instructed to complete the questionnaire independently and provide truthful responses based on their actual experiences. The data collection process yielded 654 valid responses. As shown in Table 2, 57.0% of the respondents were females. The main age group of respondents was 21–30, representing the core demographic for SHC purchases in China. The demographic profile of our sample aligns with the characteristics of the primary SHC consumer base in China, suggesting strong representativeness of the target population. This alignment enhances the practical significance and applicability of our findings to the Chinese SHC market.

**TABLE 2:** Demographic information and SHC past purchase experience of participants.

Sample characteristics	*N*	%
Gender		
Male	281	43.0%
Female	373	57.0%
**Age**		
<20	41	6.3%
21–30	357	54.6%
31–40	188	28.7%
41–50	61	9.3%
>50	7	1.1%
**Area of residency**		
Remote	309	47.2%
Regional	139	21.3%
Metropolitan	206	31.5%
**Education level**		
Secondary school or lower	58	8.9%
Pre-university or equivalent	126	19.3%
Bachelor degree	369	56.4%
Master degree or higher	101	15.4%
**Second-hand clothing purchase experience**		
Yes	192	29.4%
No	462	70.6%

### 3.3 Data analysis

Structural equation modeling was employed as the primary analytical framework due to its capacity to evaluate complex relationships among multiple interrelated variables within a comprehensive theoretical model (Wang et al., 2022). This methodology offers three distinct advantages over conventional regression techniques. First, SEM incorporates measurement error estimation through its ability to model latent constructs using multiple observed indicators, thereby enhancing measurement precision (Wang et al., 2022). Second, the simultaneous equation estimation approach effectively addresses endogeneity concerns by mitigating potential bias from reciprocal causation (Cao and Yang, 2017). Third, SEM facilitates sophisticated mediation analysis by simultaneously estimating: (a) direct effects between exogenous and endogenous variables, (b) indirect effects mediated through intervening variables, and (c) total effects encompassing both pathways (Wang et al., 2022).

To evaluate the hypothesized relationships, covariance-based structural equation modeling (CB-SEM) was employed utilizing the AMOS software. The selection of CB-SEM over variance-based approaches (e.g., PLS-SEM) was justified by the study’s primary objective of confirming a pre-specified theoretical model, in alignment with the reflective nature of the measurement constructs (Hair et al., 2017). This methodology is particularly well-suited for the assessment of overall model fit through established indices such as the comparative fit index (CFI) and the root mean square error of approximation (RMSEA). The AMOS software was selected due to its robust maximum likelihood estimation algorithm, its intuitive graphical interface for model specification, and its seamless integration with SPSS for data management. These features collectively serve to minimize operational errors and enhance analytical efficiency for complex models of this nature.

The SEM analysis comprised two complementary components (Byrne, 2016). The measurement model, which examines relationships between observed variables and their underlying latent constructs. The structural model, which specifies causal relationships among unobserved latent variables.

Data analysis was conducted using SPSS and AMOS software, following a systematic four-stage process. Preliminary analysis contains examination of demographic characteristics and assessment of scale reliability and validity using SPSS. Subsequently, confirmatory factor analysis (CFA) was employed to assess the measurement model’s convergent and discriminant validity (Anderson and Gerbing, 1988) and to verify its overall fit (Hair et al., 2010). Following this, the structural model was evaluated. This evaluation was conducted through an examination of global model fit indices (Hu and Bentler, 1999) and an analysis of the hypothesized causal pathways. Finally, invariance tests including non-restricted invariance model, full-metric invariance model, baseline model, and nested model comparisons were used to do moderation analysis.

This comprehensive analytical approach ensured rigorous examination of both the measurement properties and structural relationships specified in our theoretical framework.

## 4 Results

### 4.1 Reliability and validity analysis

The reliability and validity of the measurement scales were rigorously evaluated using SPSS software. Cronbach’s alpha coefficients ranged from 0.823 to 0.872, exceeding the recommended threshold of 0.8, while the Kaiser-Meyer-Olkin (KMO) measure of sampling adequacy yielded a value of 0.861 (Hair et al., 2012). These results demonstrate excellent internal consistency and validity across all constructs.

### 4.2 Confirmatory factor analysis (CFA)

Prior to conducting CFA, the assumption of multivariate normality was examined through a two-stage evaluation process (Kline, 2016). First, univariate statistics revealed that all scale items exhibited acceptable distributional properties, with skewness values below | 2| and kurtosis values below | 4| (Field, 2013). Second, Mardia’s standardized estimate of multivariate kurtosis was examined through the critical ratio value, confirming compliance with multivariate normality assumptions.

Then AMOS was used for confirmatory factor analysis. All factor loadings in the range of 0.725–0.811 (>0.7) and composite reliability (CR) values in the range of 0.803–0.867 (>0.7), which were both above the recommended threshold, indicating the measurement reliability, internal consistency and reliability were all good (Table 3). Moreover, the average variance extracted (AVE) values ranged from 0.578 to 0.625 (>0.5), signifying that the model had good convergent effectiveness (Bagozzi and Yi, 1988). The square root of all AVE in Table 4 were significantly greater than the relative values in their respective columns, meeting the judgment criteria of discrimination validity (Hair et al., 2017). Finally, ensuring the adequacy of SEM, we refined the model by removing items with high Chi-square values until it met fitting criteria (Segars, 1997). The χ^2^ = 1076.049, df = 663, *p* < 0.001, and the χ^2^/df value was 1.623 (<3). In addition, the fit indicators–GFI, AGFI, CFI and NNFI, values were 0.939, 0.946, 0.963, and 0.971 (>0.9), respectively. And the RMSEA value is 0.042 (<0.08), all pointing toward an excellent model fit (Rönkkö and Cho, 2022).

**TABLE 3:** Results of reliability and convergent.

Constructs and items	Loadings
Desire for gaining face: AVE = 0.578, CR = 0.803, Cronbach’s alpha = 0.826	
DGF1	0.753
DGF2	0.736
DGF3	0.785
Fear of losing face: AVE = 0.621, CR = 0.832, Cronbach’s alpha = 0.831	
FLF1	0.725
FLF2	0.795
FLF3	0.753
Attitude: AVE = 0.625, CR = 0.862, Cronbach’s alpha = 0.859	
ATT1	0.763
ATT2	0.787
ATT3	0.801
ATT4	0.794
Subjective norm: AVE = 0.592, CR = 0.831, Cronbach’s alpha = 0.823	
SN1	0.775
SN2	0.783
SN3	0.806
SN4	0.811
Perceived behavioral control: AVE = 0.614, CR = 0.843, Cronbach’s alpha = 0.872	
PBC1	0.773
PBC2	0.793
PBC3	0.803
PBC4	0.769
Purchase intention: AVE = 0.618, CR = 0.867, Cronbach’s alpha = 0.865	
PI1	0.793
PI2	0.807

**TABLE 4:** Results of discriminant reliability.

Constructs	DGF	FLF	ATT	SN	PBC	PI
DGF	0.760					
FLF	0.072	0.788				
ATT	0.175	−0.297	0.791			
SN	0.238	−0.361	0.162	0.769		
PBC	0.025	−0.075	0.057	0.078	0.784	
PI	0.272	−0.313	0.226	0.337	0.317	0.786

DGF, desire for gaining face; FLF, fear of losing face; ATT, attitude; SN, subjective norm; PBC, perceived behavioral control; PI, purchase intention.

### 4.3 Structural model

To address potential common method bias inherent in self-report questionnaire designs, variance inflation factor (VIF) was computed to assess multicollinearity among latent constructs (Ahmed et al., 2024). All VIF values ranged between 1.169 and 1.231, substantially below the conservative threshold of 2.0, thereby confirming the absence of problematic multicollinearity and ensuring the robustness of subsequent analyses.

Then, we conducted SEM analysis on the research hypothesis. Results showed our proposed integrated model had better explanatory power and higher goodness-of-fit index than TPB model in terms of SHC purchase intention. The integrated model had the superiority and a batter fit (χ^2^ = 1628.611; df = 551; *p* < 0.01; χ^2^/df = 2.956; CFI = 0.912; NNFI = 0.936; SRMR = 0.059; RMSEA = 0.062) than TPB (χ^2^ = 661.365; df = 125; *p* < 0.001; χ^2^/df = 5.291; CFI = 0.843; NNFI = 0.923; SRMR = 0.083; RMSEA = 0.073) in predicting purchase intention. Moreover, our integrated model explained 51.7% variance in this decision. In contrast, the TPB model explained 36.3% variance in SHC purchase intention whereas the integrated model combining TPB and face consciousness explained 46.6% variance in SHC purchase intention. Results back our model’s effectiveness clearly.

**FIGURE 2:**
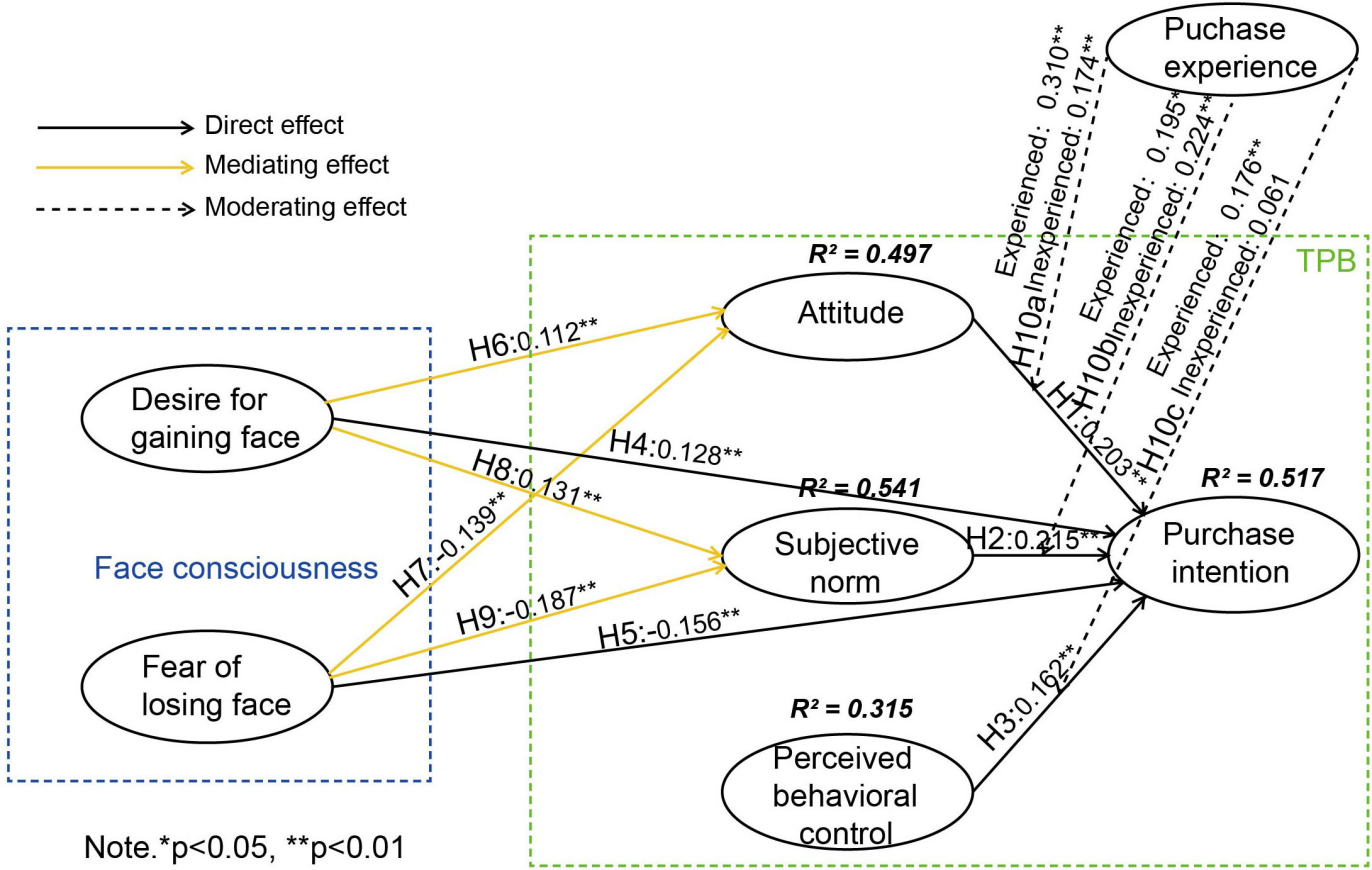
Results of the structural model.

The structural model results verified our hypotheses. Figure 2 and Table 5 show that attitude (β = 0.203, *p* < 0.001), subjective norm (β = 0.215, *p* < 0.01), and PBC (β = 0.162, *p* < 0.01) positively affected purchase intention, thus H1, H2, and H3 were all accepted. Desire for gaining face (β = 0.128, *p* < 0.001) positively influenced consumers’ SHC purchase intention, and fear of losing face (β = −0.156, *p* < 0.001) negatively influenced consumers’ SHC purchase intention. Therefore, H4 and H5 were accepted. The hypothesized effect of both desire for gaining face and fear of losing face on attitude and subjective norm were evaluated. Results revealed that desire for gaining face positively affected attitude (β = 0.112, *p* < 0.01) and subjective norm (β = 0.131, *p* < 0.001). In addition, fear of losing face negatively influenced attitude (β = −0.139, *p* < 0.01) and subjective norm (β = −0.187, *p* < 0.001).

**TABLE 5:** Path coefficients and direct effect of hypothesis.

Relations	Path coefficient	S.E.	CR	*P*-values	Result
ATT→PI	0.203	0.037	5.244	0.000	H1 accepted
SN→PI	0.215	0.042	2.783	0.006	H2 accepted
PBC→PI	0.162	0.039	5.058	0.002	H3 accepted
DGF→PI	0.128	0.041	4.146	0.000	H4 accepted
FLF→PI	−0.156	0.038	3.432	0.000	H5 accepted
DGF→ATT	0.112	0.045	3.263	0.003	–
FLF→ATT	−0.139	0.040	3.932	0.005	–
DGF→SN	0.131	0.046	3.352	0.000	–
FLF→SN	−0.187	0.025	4.467	0.000	–
Good-of-fit statistics for the integrated model χ^2^ = 1628.611 (df = 551, *p* < 0.01), CFI = 0.912, NNFI = 0.936, SRMR = 0.059, RMSEA = 0.062		Total variance explained: R^2^ of ATT = 0.497 R^2^ of SN = 0.541 R^2^ of PBC = 0.315 R^2^ of PI = 0.517		Total effect on purchase intention: DGF = 0.181 FLF = −0.226 ATT = 0.203 SN = 0.215 PBC = 0.162	

DGF, desire for gaining face; FLF, fear of losing face; ATT, attitude; SN, subjective norm; PBC, perceived behavioral control; PI, purchase intention.

Subsequently, we further study the indirect effect of constructs. Results (Table 6) indicated that desire for gaining face (β = 0.053, *p* < 0.05) and fear of losing face (β = −0.070, *p* < 0.01) had an indirect effect on purchase intention. Therefore, H6, H7, H8 and H9 were accepted. The above results suggest that the constructs in our integrated framework had an important mediating effect on the formation of intention.

**TABLE 6:** Standardized indirect-effect.

Indirect effect of	On
	PI
DGF	0.053*
FLF	−0.070**

DGF, desire for gaining face; FLF, fear of losing face; PI, purchase intention. *P < 0.05, **p < 0.01.

### 4.4 Non-restricted and full-metric invariance models

To evaluate the hypothesized moderating role of past purchase experience in our integrated model, a series of measurement invariance tests were conducted. The sample was stratified into two distinct groups: 462 participants without prior SHC purchase experience and 192 participants with such experience.

The analysis commenced with the establishment of a non-restricted baseline model, which demonstrated acceptable fit indices (χ^2^ = 2652.174, df = 1358, *p* < 0.001, RMSEA = 0.061; CFI = 0.908; IFI = 0.912). Subsequently, a full-metric invariance model was specified, constraining all parameters to be equal across groups while maintaining good model fit (χ^2^ = 2686.146, df = 1388, *p* < 0.001, RMSEA = 0.061; CFI = 0.901; IFI = 0.907). The critical comparison between these nested models revealed no statistically significant difference [Δχ^2^(30) = 33.972, *p* > 0.01], supporting the establishment of full metric invariance. This finding indicates that the measurement properties of our constructs remain equivalent across groups with differing purchase experiences. Complete results of the measurement invariance analysis are presented in Table 7.

**TABLE 7:** Results of measurement and structural-invariance models.

Measurement-invariance models for experienced (*N* = 192) and inexperienced (*N* = 462) in SHC purchase							
Models		χ^2^	df	Δ χ^2^		Full-metric invariance	
Non-restricted model		2652.174	1358	Δχ^2^ (30) = 33.972, *p* > 0.01 (not significant)		Accept	
Full-metric invariance		2686.146	1388				
Other Goodness-of-fit indices of the non-restricted model: RMSEA = 0.061;CFI = 0.908; IFI = 0.912							
Other Goodness-of-fit indices of the full-metric invariance: RMSEA = 0.061;CFI = 0.901; IFI = 0.907							
Structural-invariance model for experienced (*N* = 192) and inexperienced (*N* = 462) in SHC purchase							
**Paths**	**Experienced**		**Inexperienced**		**Baseline model (freely estimates)**		**Nested model (constrained to be equal)**
	**Coefficients**	***P*-values**	**Coefficients**	***P*-values**			
ATT←PI	0.310	0.006	0.174	0.009	χ^2^ (1346) = 2425.367		χ^2^ (1347) = 2433.437^a^
SN←PI	0.195	0.035	0.224	0.007	χ^2^ (1346) = 2425.367		χ^2^ (1347) = 2425.630^b^
PBC←PI	0.176	0.002	0.061	0.095	χ^2^ (1346) = 2425.367		χ^2^ (1347) = 2432.356^c^
Chi-square difference test:				Other goodness-of-fit indices of the baseline model:			
^a^Δχ^2^ (1) = 8.070, *p* < 0.05 (H10a-accepted)				RMSEA = 0.069;CFI = 0.900; IFI = 0.906			
^b^Δχ^2^ (1) = 0.263, *p* > 0.05 (H10b-rejected)							
^c^Δχ^2^ (1) = 6.989, *p* < 0.01 (H10c-accepted)							

ATT, attitude; SN, subjective norm; PBC, perceived behavioral control; PI, purchase intention.

^a^The constraint of equality on the path coefficients ATT→PI.

^b^The constraint of equality on the path coefficients SN→PI.

^c^The constraint of equality on the path coefficients PBC→PI.

### 4.5 Structural-invariance models

A baseline model was established by incorporating structural relationships among study variables into the full-metric invariance framework. The baseline model demonstrated excellent fit to the empirical data (χ^2^ = 2425.367, df = 1346, *p* < 0.01; RMSEA = 0.069; CFI = 0.900; IFI = 0.906).

To examine the hypothesized moderating effects, this baseline model was systematically compared with a series of nested models in which specific structural paths were constrained to equality across groups with and without SHC purchase experience. The invariance testing revealed significant findings. A statistically significant difference emerged in the relationship between attitude and purchase intention across consumer groups [Δχ^2^(1) = 8.070, *p* < 0.05], supporting H10a. No significant difference was found for the path between subjective norm and purchase intention [Δχ^2^(1) = 0.263, *p* > 0.05], leading to the rejection of H10b. The association between PBC and purchase intention differed significantly between groups [Δχ^2^(1) = 6.989, *p* < 0.01], confirming H10c. These results, comprehensively presented in Figure 2 and Table 7, demonstrate that past purchase experience serves as an important moderator in the formation of SHC purchase intentions, particularly influencing the effects of attitude and perceived behavioral control.

## 5 Discussion and implications

### 5.1 Discussion

The analysis reveals distinct patterns in SHC purchase intention among Chinese consumers. Notably, subjective norm demonstrates stronger predictive power than attitude within the TPB framework. This finding contrasts with previous research (Cao and Yang, 2017) and likely reflects Chinese collectivist cultural context, where social influences outweigh individual attitudes in consumption decisions.

The dual dimensions of face consciousness emerge as significant predictors, with the desire for gaining face positively influencing SHC evaluations while fear of losing face exerts negative effects. These results findings are consistent with prior research emphasizing positive face motivations (Wei and Jung, 2017; Chen K. et al., 2019) but contrast with studies that focus on negative face concerns, such as the work by Wang et al. (2018). This discrepancy is likely attributable to a fundamental difference in research context. The study by Wang et al. (2018) was situated within the domain of energy-saving behaviors, wherein reduced consumption constitutes the pro-environmental action. In this context, face consciousness was shown to promote a status-seeking lifestyle, thereby discouraging energy-saving behaviors. Conversely, the present study examines SHC consumption, which itself represents a form of sustainable consumption. Within this context, sustainable consumption is adopted as a means to enhance one’s social reputation and image. Furthermore, although Wei and Shen (2025) considered the effects of both “gain face” and “avoid losing face” on green consumption behavior simultaneously, they both have positive effects on green consumption behavior. The unique characteristics of SHC - simultaneously symbolizing environmental consciousness and potential social stigma - likely explain these divergent findings compared to general green consumption research.

The results indicate that attitude and subjective norm mediate the relationship between the two dimensions of face consciousness and the intention to purchase SHC products. This finding suggests that face consciousness serves as a significant antecedent of both attitude and subjective norm, a conclusion that is consistent with prior research by Li and Su (2007) and Long and Abd Aziz (2022).

Regarding the moderating effect of past purchase experience on consumers’ SHC purchase intention, results showed that past purchase experience exerts an important moderator on the SHC purchase intention through the TPB. This result is consistent with the finding of Koay et al. (2022). However, the influence of subjective norm on purchase intention is insignificantly different between experienced and non-experienced consumers, which is inconsistent with the findings of Koay et al. (2022). The primary reason for this discrepancy lies in differences in research contexts. Koay et al. (2022) focused on intention to engage in digital piracy, whereas this study concentrated on SHC purchase intention. Compared with the former, clothing presents one’s image more directly. So, regardless of whether there is a purchase experience or not, the influence of subjective norm on intention is significant. Furthermore, the finding that past purchase experience does not moderate the relationship between subjective norm and purchase intention. Although prior experience typically empowers people to rely more on personal knowledge than on external social cues (Chi-Ming et al., 2016), this tendency was not observed within the context of SHC consumption. This suggests that the influence of social norms may be so potent in this domain that their impact remains undiminished even in the presence of direct personal experience.

### 5.2 Theoretical implications

This study makes several significant theoretical contributions to the literature on SHC consumption. The TPB has been widely applied to predict purchase intention for various green products (Kian et al., 2022; Madeline et al., 2023; Tryphena and Arul Aram, 2023). However, existing literature indicates that critical gaps persist in emerging consumption contexts. Notably, few studies have integrated cultural factors with TPB to examine consumers’ SHC purchase intentions (ElHaffar et al., 2020). To address this gap, the current study responds to longstanding criticisms of TPB’s overreliance on rational decision-making by incorporating the cultural dimension of face consciousness (both desire for gaining face and fear of losing face) alongside experience-based factors.

Structural model comparisons revealed that integrating desire for gaining face, fear of losing face, past purchase experience, attitude, subjective norm, and PBC into a unified framework effectively explains the formation of consumers’ SHC purchase intention. The proposed theoretical model offers greater comprehensiveness and explanatory power in predicting purchase intention, with potential extensions to other sustainable consumption behaviors. Furthermore, structural invariance testing demonstrated that the effects of attitude and PBC on purchase intention are moderated by consumers’ past purchase experiences. From a theoretical standpoint, this study provides critical validation for the necessity of incorporating consumers’ prior purchase experience when analyzing SHC purchase intention.

### 5.3 Practical implications

The findings yield several important implications for enterprises and policymakers, though these should be considered in light of the study’s methodological limitations. As this research employed a cross-sectional design with self-reported data from Chinese consumers, and the cultural specificity of face consciousness constructs suggests these implications may be most directly applicable to similar cultural contexts. Practitioners should view these recommendations as empirically-informed starting points for strategy development that require validation through market testing and adaptation to local conditions.

First, attitude directly affects consumers’ SHC purchase intention and mediates the relationship between face consciousness and purchase intention, suggesting that marketing strategies could consider emphasizing cultivating positive attitude toward SHC. This might be achieved by highlighting the multidimensional value proposition of SHC–including environmental benefits and emotional appeal–through digital marketing channels (Kian et al., 2022). Concurrently, potential consumer concerns, particularly perceived risks associated with second-hand fashion consumption, should be proactively addressed (Kian et al., 2024).

The significant influence of subjective norm indicates that marketing strategies might leverage social networks to amplify positive word-of-mouth (Tu and Hu, 2018). Given the pervasive role of social media in shaping consumer behavior, SHC brands and government agencies could consider encouraging existing customers and influencers to share favorable experiences (Joshi et al., 2025), thereby potentially fostering a social environment conducive to SHC adoption.

The positive impact of PBC suggests that second-hand fashion brands should diversify sales channels, offering SHC not only in traditional brick-and-mortar stores but also through online platforms^2^.

The positive impact of PBC suggests that second-hand fashion brands may want to diversify sales channels, offering SHC not only in traditional brick-and-mortar stores but also through online platforms^2^.

Both the desire for gaining face and the fear of losing face were found to directly influence SHC purchase intention. Given the cultural specificity of these constructs, these strategies should be particularly tested in similar cultural contexts. Based on these findings, SHC brands could consider two strategic approaches: first, product quality might be enhanced to ensure that consumption is associated with face-gaining rather than face-losing outcomes, for instance, by offering high-quality, fashionable, and well-crafted products (Ferraro et al., 2016). Second, targeted marketing campaigns could be implemented to strengthen the association between SHC purchases and face-gaining attributes (e.g., by promoting the uniqueness and environmental friendliness of SHC) (Kian et al., 2022, 2023), or to weaken the association with face-losing perceptions (e.g., by countering the notion that SHC products are cheap or of low status).

The moderating role of past purchase experience underscores the potential need for SHC brand managers to segment consumers based on prior engagement. Understanding how purchase intentions are shaped by attitude and PBC across different consumer groups might enable marketers to tailor engagement strategies accordingly. Conversely, the finding that past purchase experience does not moderate the relationship between subjective norm and purchase intention suggests that strategies leveraging social influence–such as the use of testimonials, influencer partnerships, and the highlighting of social approval–are likely to be effective uniformly across both novice and experienced consumer segments. Consequently, marketing communications could consistently emphasize the social acceptance and increasing prevalence of SHC consumption to effectively target the entire market.

We recommend that organizations implement these suggestions as pilot programs with careful measurement of outcomes before full-scale implementation, particularly when applying them in cultural contexts different from the Chinese market studied here.

## 6 Conclusions, limitations and future research

### 6.1 Conclusion

The purpose of this study was to establish an integrated theoretical framework for the comprehensive examination of factors influencing Chinese consumers’ SHC purchase intention. This was achieved by integrating cultural factors, specifically face consciousness, and past purchase experience into the TPB, thereby extending existing integrative models. The proposed framework was generally supported by the empirical results. Hypothesis testing revealed that enhancing attitude, PBC, and subjective norm can effectively strengthen consumers’ intentions to purchase SHC products. Furthermore, both the desire for gaining face and the fear of losing face were found not only to directly affect purchase intention but also to exert an indirect influence through the mediating roles of attitude and subjective norm. Additionally, past purchase experience was identified as a significant moderator in the formation of SHC purchase intention, particularly influencing the paths from attitude and PBC. This research contributes to the further enrichment of literature pertaining to environmental sustainability, economic sustainability, and consumer behavior. Moreover, it offers actionable insights for marketing practitioners and provides a strategic blueprint for strategies aimed at enhancing SHC purchase intention.

### 6.2 Limitations and future research

This study provides significant insights into the drivers of SHC purchase intention within the Chinese consumer market. However, several limitations must be acknowledged, which also present avenues for future research. Primarily, due to budgetary constraints, the investigation was confined to the socio-cultural context of China and focused specifically on SHC products. Consequently, the findings and the proposed model relationships are inherently culturally bounded, and their direct generalizability to other cultural settings or to other second-hand product categories may be limited. It is, therefore, advised that the extrapolation of these findings to other contexts be approached with caution pending further validation. To establish the boundary conditions and assess the cross-cultural validity of the model, future studies are strongly encouraged to conduct replications across diverse cultural settings and with varied product categories.

Second, the data in this study were primarily derived from participant self-reports at a single point in time. Consequently, the findings represent static respondent feedback and lack insights into dynamic behavioral changes. It is recommended that subsequent research employ alternative methodological approaches, such as longitudinal designs, case studies, in-depth qualitative interviews, or grounded theory methodology, to further elucidate the influence of face consciousness on consumers’ purchase intention.

Third, the current study did not account for several contextual and psychological variables known to influence SHC consumption decisions. Key contextual factors, such as product quality, information quality, and service quality (Olga et al., 2024; Susana et al., 2021), were not considered. Additionally, relevant psychological constructs, including perceived risk, perceived value, and effort expectancy (Jun et al., 2022; Kian et al., 2023; Zahid et al., 2023) were omitted from the analytical framework. Future studies should integrate these variables to develop a more nuanced understanding of the determinants of SHC purchase intentions.

## Data Availability

The raw data supporting the conclusions of this article will be made available by the authors, without undue reservation.

## Footnotes

1
www.wenjuan.com

2 Taobao, JD.com
